# NEDD9 sustains hexokinase expression to promote glycolysis

**DOI:** 10.1038/s41389-022-00391-w

**Published:** 2022-04-11

**Authors:** Alexander Y. Deneka, Anna S. Nikonova, Hyung-Ok Lee, Warren D. Kruger, Erica A. Golemis

**Affiliations:** 1 Program in Molecular Therapeutics, Fox Chase Cancer Center, Philadelphia, PA 19111 USA; 2 Lewis Katz School of Medicine at Temple University, Philadelphia, PA 19140 USA

**Keywords:** Non-small-cell lung cancer, Glycobiology

## Abstract

Elevated rates of glycolysis in cancer cells support tumor growth, in a process that typically depends on oncogene-induced increases in the expression and/or activity of enzymes in the glycolytic pathway. The NEDD9 scaffolding protein is upregulated in many advanced tumors, with increased NEDD9 promoting the activity of SRC and other effectors that promote invasion and metastasis. We here define a new role for NEDD9 in support of glycolysis. NEDD9 knockdown significantly impaired glycolysis in multiple lung cancer cell lines This was accompanied by post-transcriptional downregulation of steady-state levels of hexokinases (HK1 and HK2), which catalyze early steps in the glycolytic cascade, key rate limiting enzyme phosphofructokinase (PFK1), and downstream glyceraldehyde phosphate dehydrogenase (GAPDH). In mice, protein levels of HK1, HK2, PFK1, and GAPDH were depressed in *Kras*^*tm4Tyj/J*^ */Trp53*^*tm1Brn/J*^ (*KP*) non-small cell lung tumors with null versus wild type *Nedd9*. Reciprocally, depletion of HK1 or HK2 elevated NEDD9 expression, as did the treatment of cells with 2-deoxyglucose (2DG), an inhibitor of glycolysis; whereas overexpression of hexokinases promoted NEDD9 dephosphorylation, associated with reduced NEDD9 activity. Together, these data for the first time suggest a negative feedback circuit involving NEDD9 and glycolytic enzymes that may contribute to NEDD9 action in promoting the aggressive growth of advanced tumors.

## Introduction

Tumorigenesis is accompanied and supported by the Warburg effect; a process in which cancer cells increase their rates of glycolysis, even in circumstances in which intact mitochondria allow for more energy-efficient production of ATP through oxidative phosphorylation. The increased dependence on glycolysis contributes to robust tumor growth in multiple ways, including enhanced production of additional metabolites such as NADPH and acetyl-CoA that are important for nucleic acid, protein, and fatty acid biosynthesis [1, 2]. The selective requirement for glycolysis in cancer cells has suggested inhibition of this process might offer a promising tumor-selective target for therapy, and spurred intensive efforts to study the mechanisms by which it is regulated. This has led to the recognition that activation of specific oncogenes [3, 4] or loss of tumor suppressors [5, 6] increases glycolysis by increasing the expression and activity of enzymes in the glycolytic pathway.

Retrovirally-borne v-SRC was one of the first identified transforming oncogenes [7, 8], and suggestively, in early studies, cells transformed with v-Src were reported to have elevated expression of glycolytic enzymes [9, 10]. In sporadic human cancers, c-SRC activity is typically enhanced based on c-SRC conformational changes [11] induced by partner proteins including members of the CRK-associated substrate (CAS) family proteins [12, 13]. CAS proteins are non-catalytic, but serve as scaffolding proteins, assembling complexes between SRC, FAK, and downstream effector proteins. Among these SRC activating CAS proteins, NEDD9 (alternatively designated HEF1 or CAS-L) is notable because it is strongly upregulated during tumor progression [14, 15], promoting invasion and metastasis by stimulating SRC and FAK phosphorylation of substrates that promote cytoskeletal rearrangement, cell migration, and survival signaling [16]. Conversely, loss of NEDD9 reduces SRC activation in many settings [17–19].

No prior study has addressed a potential link between NEDD9 and cellular dependence on glycolysis. However, the SRC-NEDD9 signaling relationship suggested the novel hypothesis that NEDD9 expression may play a role in modulating glycolysis, potentially through controlling activity of SRC. In this study, we demonstrate that depletion of NEDD9 severely impairs cellular capacity for glycolysis, and causes multiple defects in the expression and activation of enzymes in this pathway, both in vitro and in vivo.

## Results

### NEDD9 depletion impairs glycolytic capacity in human and murine non-small cell lung cancer (NSCLC) cell line models

**Fig. 1:**
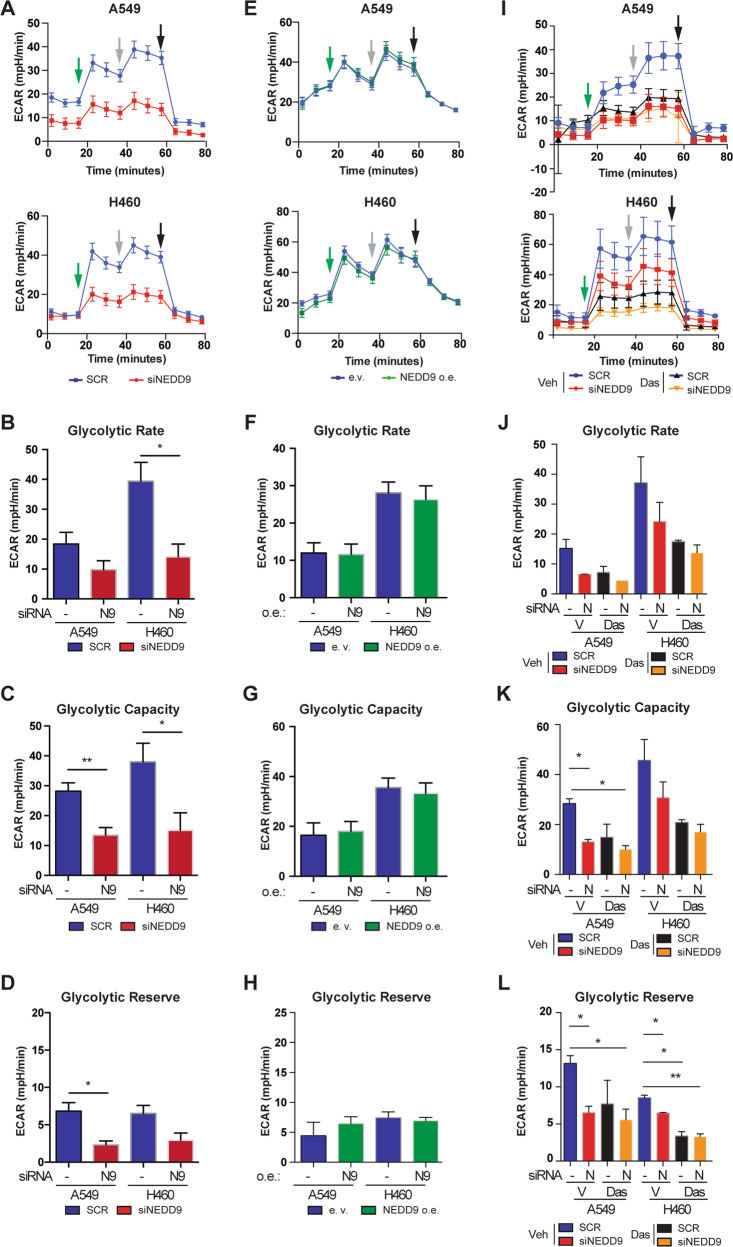
Depletion of NEDD9 in human NSCLC cell lines impairs glycolysis. **A** Seahorse results in A549 and H460 cells treated with siRNA to NEDD9 (red) or scrambled siRNA (SCR, blue). Glycolysis is determined by measuring lactic acid acidification of the media. Arrows indicate addition of glucose (green), oligomycin (gray), and 2DG (black). **B**–**D**. Quantitation of results from (**A**) to demonstrate differences in glycolytic rate (**B**), glycolytic capacity (**C**), and glycolytic reserve (**D**). **E**–**H** Experiments parallel those shown in (**A**–**D)**, but in conditions of 48 h of overexpressed NEDD9 (green) contrasted to cells transfected with empty vector (e.v., blue). **I**–**L** Experiments parallel those in (**A**–**D**), but in cells treated with vehicle or dasatinib. ECAR, extracellular acidification rate. Graphs shown represent average of three independent experiments. *p < 0.05, **p < 0.01; error bars represent SEM.

To establish whether changes in NEDD9 expression influenced cellular capacity for glycolysis, we used a Seahorse assay to directly assess the functional consequence of depleting or overexpressing NEDD9 [20] (Fig. 1). In this 3-step approach, the addition of glucose to the cell culture media triggers basal glycolysis; subsequent addition of oligomycin triggers cells to their maximal glycolytic capacity by blocking mitochondrial ATP generation; finally, addition of 2-deoxy-glucose terminates glycolysis by competing with glucose for binding to hexokinase. Two days after depletion of NEDD9 (Fig S1A), the capacity of cells to perform glycolysis was markedly reduced in four independent human and murine NSCLC cell lines (Fig. 1A, S1B). Segmentation of the Seahorse data shows normal basal rates of glycolysis in 3 of the 4 cell lines, and slight depression in 1 cell line (Fig. 1B, S1C). However, response to glucose and maximum glycolytic capacity, were strongly reduced or eliminated in all of the NEDD9-depleted cell lines (Fig. 1C, S1D), and glycolytic reserve (the difference between maximum glycolytic capacity and basal glycolytic rate) was also reduced (Fig. 1D, S1E). These results indicated endogenous levels of NEDD9 were required for efficient response to glucose. In contrast to results with NEDD9 depletion, overexpression of NEDD9 (Fig S1A) did not affect glycolysis, indicating endogenous levels were saturating for biological effect (Fig. 1E–H, S1F–I).

To assess the relationship of NEDD9 and SRC, we performed parallel experiments in the A549 and H460 cell lines, in which we treated cells with vehicle or with the SRC inhibitor dasatinib for three hours before performing Seahorse analysis (Fig. 1I–L). These experiments indicated that depleting NEDD9 was comparable to inhibition of SRC kinase activity in its effect on glycolysis. It also indicated that treatment of NEDD9-depleted cells with dasatinib did not result in additional statistically significant reduction in glycolysis. In complementary work, we stimulated SRC activation with EGF (as in [21]) in NEDD9-depleted or control depleted cells, and repeated Seahorse analysis. EGF stimulation did not reverse the reduced glycolytic capacity of cells with reduced NEDD9, even though SRC activity was enhanced; we note, NEDD9 depletion was also associated with reduced levels of total SRC (Fig S2).

### Nedd9 depletion impairs expression of glycolytic enzymes and glycolytic capacity in human NSCLC cell lines

**Fig. 2:**
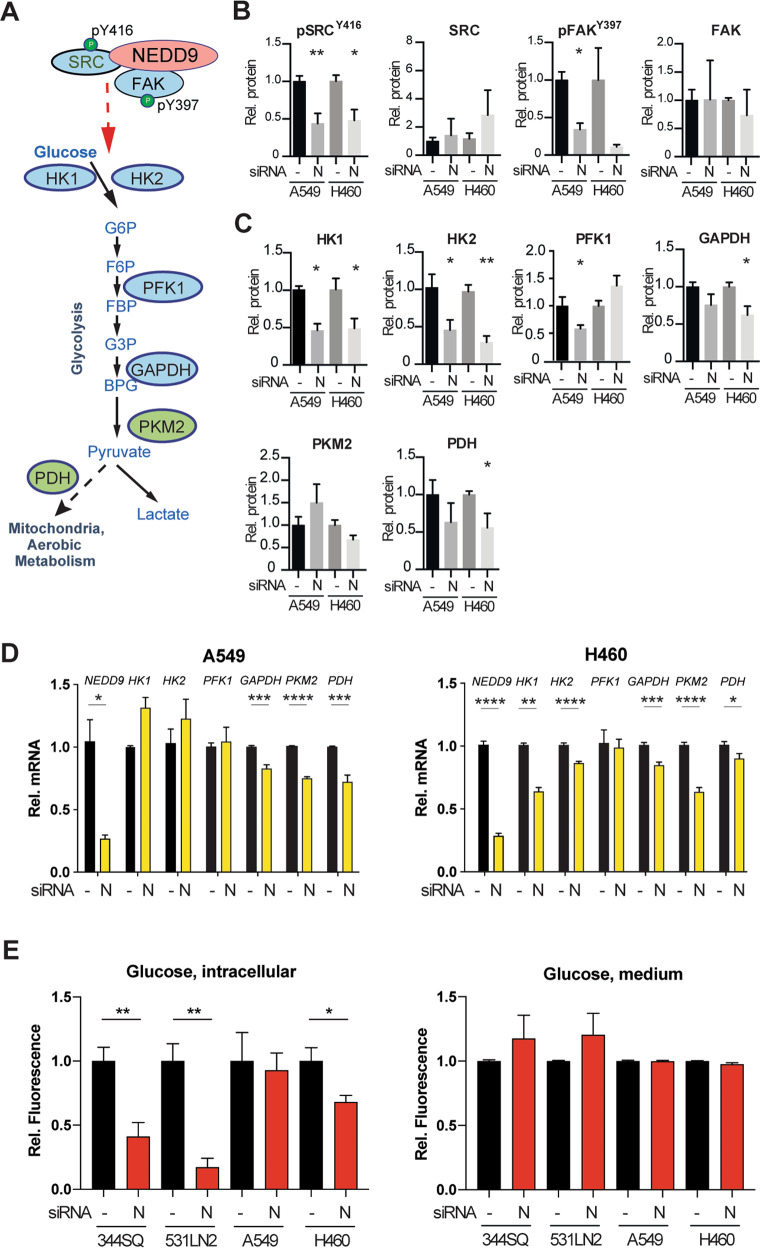
Depletion of NEDD9 in NSCLC cells reduces expression and activity of hexokinases and other glycolytic enzymes. **A** Schematic of proteins that regulate and mediate glycolysis. **B** Western blot analysis of total and active phosphorylated (pSRC^Y416^, pFAK^Y397^) SRC and FAK following treatment with siRNAs depleting NEDD9 (N) protein or Scrambled control (-). **C** Western blot analysis of proteins indicated, following NEDD9 depletion. **D** qRT-PCR analysis of steady-state mRNA for genes indicated in NEDD9-depleted (N) or scrambled siRNA-treated (-) cells. **E** Levels of intracellular glucose (left) and glucose in culture medium (right) in cells treated with siRNAs depleting NEDD9 (N) or Scrambled control (-). Data are normalized for each cell line to cells treated with Scr siRNA. *p < 0.05, **p < 0.01; error bars represent SEM.

To gain insight into the mechanism by which NEDD9 depletion reduced glycolysis, we siRNA depleted NEDD9 expression in the A549 and H460 NSCLC cell lines for 48 h, then probed the expression and activity of the NEDD9 effectors FAK and SRC, and of proteins involved in glycolysis (Fig. 2A). After 48 h, depletion of NEDD9 caused the expected reduction in activation of the NEDD9 partner proteins SRC (ph^Y416^SRC) and FAK (ph^Y397^FAK) (Fig. 2B, S3A). In addition, depletion of NEDD9 very significantly reduced protein expression of HK1 and HK2, but had modest or no effects on the expression of other proteins downstream in the pathway including PFK1, GAPDH, PKM2, and PDH (Fig. 2C, S3B). Parallel analysis by qRT-PCR indicated NEDD9 depletion did not affect steady-state mRNA expression of hexokinases in A549 cells, and slightly reduced hexokinase mRNA expression in H460 cells, albeit not sufficient to explain the effect on protein levels of HK1 and HK2 (Fig. 2D). These results implied that NEDD9 depletion reduced hexokinase expression in part through post-transcriptional mechanism of regulation. Quantitatively minor but significant reduction in mRNA expression of *GAPDH*, *PKM2*, and *PDH* were also observed, and correlated with insignificant changes in protein expression.

We also investigated the consequences of NEDD9 overexpression in these cell models (S3C, D). We found overexpression induced activation of its direct partner SRC, as anticipated [22]; however, it did not induce expression of any of the enzymes in the glycolytic pathway. Overall, these data indicate that the NEDD9 protein is necessary to support protein expression of hexokinases acting at the top of the glycolytic pathway, but no further gain in expression of these enzymes is induced by exceeding endogenous levels of NEDD9.

### Genetic ablation of Nedd9 reprograms expression of enzymes required for glycolysis in murine model

**Fig. 3:**
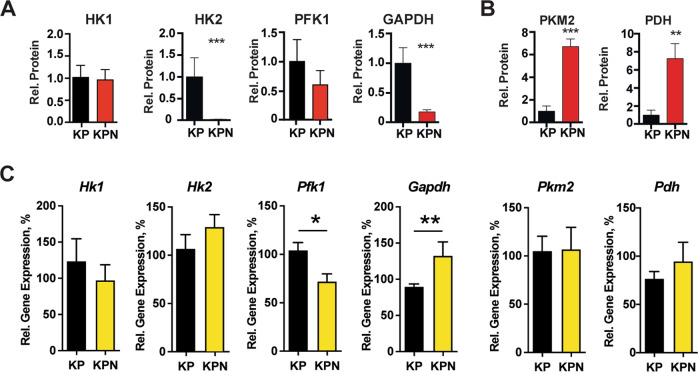
Altered expression of glycolytic pathway enzymes in *KPN* versus *KP* murine NSCLC tumors. **A**, **B** Quantification of Western blots indicating reduced (**A**) or increased (**B**) expression of expression of the indicated glycolytic pathway enzymes in *KPN* versus *KP* tumors. **C** qRT-PCR showing steady-state mRNA expression of indicated genes in *KPN* versus *KP* tumors analyzed in (**A**, **B**). *p < 0.05, **p < 0.01, ***p < 0.001, ****p < 0.0001 for all graphs.

To complement studies of consequences of transient depletion of NEDD9 for expression of effectors and glycolytic pathway enzymes, we also examined their expression in biological specimens from NSCLC tumors that formed in mice with inducible, lung-specific activation of *Kras* and loss of *Trp53* (*Kras*^*tm4Tyj/J*^ */Trp53*^*tm1Brn/J*^ [23, 24] mice) with a wild type versus a null allele for *Nedd9* [25] (*KP* versus *KPN* mice) (Fig. 3, S4). In previous work, we determined that *KPN* mice had greatly reduced activation of SRC [17]. As with cells with NEDD9-depleted in vitro (Fig. 2), *KPN* tumors also had an almost total loss of HK2, and strong reductions in phosphofructokinase (PFK1), and GAPDH (Fig. 3A). In contrast, the *KPN* genotype was associated with elevated expression of pyruvate kinase M2 (PKM2), and increased pyruvate dehydrogenase (PDH), operating downstream in the glycolytic cascade (Fig. 3B). Measurement of steady-state mRNA expression in tumor lysates either detected no significant differences in expression of these genes discriminating the *KP* and *KPN* genotypes, or indicated changes in expression opposite to that observed with encoded proteins (Fig. 3C). These results also suggest a post-transcriptional response to the loss of *Nedd9*. Overall, the reduction in expression of enzymes catalyzing early steps in the glycolytic cascade paired with the elevation of expression of enzymes catalyzing downstream steps suggested a primary consequence of NEDD9 deficiency was the restriction of entry into the pathway by targeting hexokinase 2 (as observed in transient experiments, Fig. 2) and additional upstream elements of the glycolytic cascade, followed by compensating upregulation of downstream pathway components.

### NEDD9 expression and phosphorylation are regulated by hexokinase expression and activity of the glycolytic pathway

**Fig. 4:**
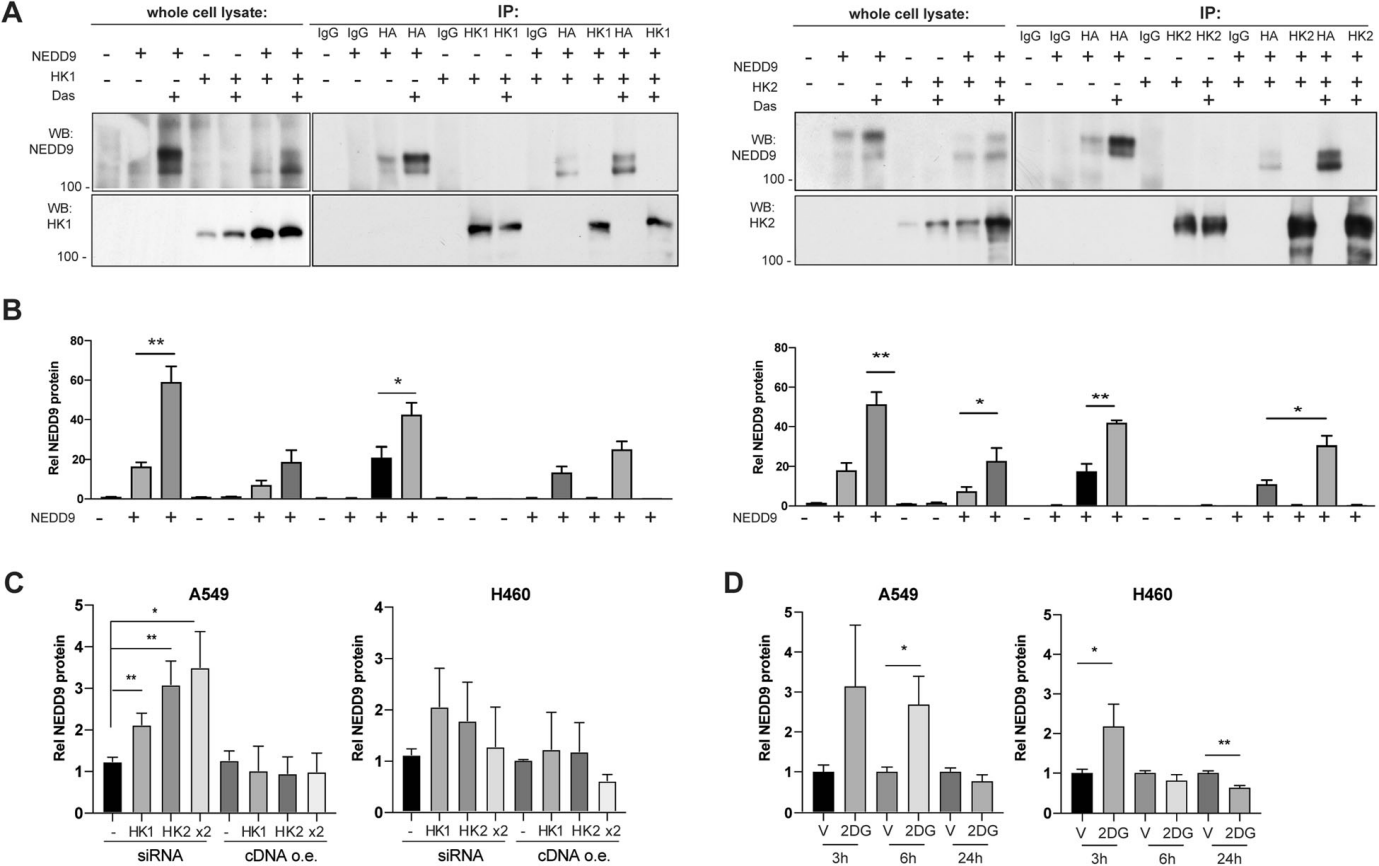
Hexokinase depletion and 2DG treatment induce NEDD9 expression. **A**, **B** Western blot of whole cell lysates or immunoprecipitates of HEK293 cells overexpressing empty vectors (-), HA-NEDD9, or GFP-HK1 (HK1; left panel), or GFP-HK2 (HK2; right panel) as indicated. Shown, representative experiments (**A**), and quantitation of results from multiple experiments (**B**). **C** Western blot analysis of NEDD9 expression in NSCLC cell lines with depletion (siRNA) or overexpression (o.e.) of control (-), HK1, HK2, or both (x2). **D** Western blot quantification of NEDD9 expression in cells treated with vehicle (V) or 2DG for times indicated. All quantification is based on at least three independent experiments, with data normalized to Ponceau staining. *p < 0.05, **p < 0.01, for all graphs.

Given NEDD9 influenced hexokinase expression at a post-transcriptional level, we used co-immunoprecipitation to determine if NEDD9 directly associated with HK1 or HK2, based on co-expression of co-overexpressed HA-NEDD9 and GFP-fused-HK1 or -HK2 in HEK293 cells. We were unable to detect association, whether antibodies to HA or to the hexokinases were used for immunoprecipitation (Fig. 4A). Some NEDD9 protein interactions are regulated by SRC phosphorylation of NEDD9 [16]; however, treatment with dasatinib to inhibit SRC did not promote co-immunoprecipitation between NEDD9 and HK1 or HK2, although it did increase expression both of NEDD9 and of hexokinases (Fig. 4A).

In co-expression experiments, we noted that co-overexpression of either HK1 or HK2 with NEDD9 in HEK293 cells was associated in increased NEDD9 expression, reduction in the slower migrating (hyperphosphorylated) 115 kDa form of NEDD9, and a proportionate increase in the faster migrating 105 kDa protein form (Fig. 4A, B). This suggested reciprocal signaling from glycolytic pathway enzymes to control of NEDD9 phosphorylation. Prior studies have demonstrated that the 115 kDa form of NEDD9 is less stable, although it is also associated with greater NEDD9 activity as a signaling intermediate [26–28]. Extending this observation, we depleted HK1, HK2, or both in A549 and H460 cells, and determined that this resulted in increased NEDD9 protein expression (Fig. 4C, S5A), without commensurate changes in NEDD9 mRNA (Fig S5B). We then directly tested whether inhibition of glycolysis by treatment with 2DG for 3, 6, or 24 h affected NEDD9 expression or phosphorylation. In both A549 and H460 cells, NEDD9 expression significantly increased within 3-6 h of 2DG treatment (Fig. 4D, S5C).

## Discussion

This article for the first time demonstrates that NEDD9 supports glycolysis in lung cancer cells and tumors. Transient depletion of NEDD9 reduces the rate of glycolysis and glycolytic reserve, and is associated with a significant reduction in levels of HK1, HK2, and/or PFK1 in vitro. The magnitude of this reduction was similar to that observed following use of an inhibitor of c-SRC, a NEDD9 partner kinase, and may be mediated by NEDD9 control of c-SRC activity. Reciprocally, inhibition of glycolysis by treatment with 2DG and transient depletion of hexokinases elevated NEDD9 expression and phosphorylation. Supporting physiological relevance during tumorigenesis, reductions in HK1, HK2, and PFK1 were observed in lung tumors that formed in the absence of Nedd9, in *KPN* mice. These data offer new insight into the importance of NEDD9 in supporting the growth of invasive and metastatic human tumors, previously attributed largely to effects on classic cell attachment and proliferative signaling pathways, and on cell cycle controls [16, 29, 30].

HK2 plays a critical role in *KRAS-*dependent tumorigenesis in mouse lung cancer models, and in other cancers, with loss of HK2 attenuating tumor phenotypes and improving survival [31]. Interestingly, although loss of *Nedd9* in mouse models for other tumor types also slows tumor progression and improves survival (e.g. [18, 32]), in the *KPN* mice analyzed in this study (Fig. 3), tumor growth was enhanced by loss of NEDD9 [17]. In reconciling these data, we note that lung tumors are uniquely dependent on autophagy [33], *KPN* tumors had highly elevated autophagy relative to *KP* tumors, and that the enhanced growth phenotype of *KPN* tumors versus *KP* tumors was completely eliminated by treatment with the autophagy blocker chloroquine [17]. We hypothesize that the increase in autophagy provided a pro-growth stimulus that countered the reduced growth that would be conferred by reduced HK2 activity. We also note that while the expression of enzymes at the top of the glycolytic pathway is reduced, the expression of enzymes operating downstream, including PKM2 and PDH, is sharply elevated in tumor tissues in vivo, further buffering the biological consequences of reduced availability of intermediate metabolites. This likely reflects compensation for the constitutive loss of NEDD9. In contrast, we observed cell lines with transient reduction of *NEDD9* were unable to sufficiently upregulate PDH and PKM, in accord with the striking defect in glycolysis in these cells. While the Seahorse assay provides an overall indication that cell growth in the absence of NEDD9 leads to an altered response to glucose, a more detailed biochemical analysis of the role of NEDD9 in regulating the expression and activity of intermediate enzymes would be an important next step.

We hypothesize that the most likely mechanism by which loss of NEDD9 influences glycolysis is through impairing the activation and functionality of c-SRC, particularly as prior studies have documented a direct role of SRC phosphorylation of hexokinases in stabilizing these proteins [21]. These data are in accord with our results demonstrating the effect of NEDD9 loss on HK1, HK2, and PFK1 is post-transcriptional. A growing number of studies have identified SRC kinase activity as important for controlling the activity of the glycolytic pathway, including studies finding SRC phosphorylation directly or indirectly regulates the activity of LDHA [34] and PKM2 [35]. The facts that dual dasatinib treatment and NEDD9 activation do not further reduce glycolytic capacity, while EGF stimulation of SRC in the context of NEDD9 depletion does not restore glycolytic capacity, are compatible with the idea that NEDD9 contributes to SRC stabilization and potentially engagement with the hexokinases, reflecting its well-known scaffolding function for SRC interactions [36]. Whether NEDD9 additionally modulates the activity of SRC in controlling the activity of these downstream glycolytic enzymes remains to be explored.

Finally, NEDD9 has variable expression in lung tumors, based on increased transcription in a subset of typically advanced tumors, coupled with signaling changes that influence protein stability (for instance, in TGFβ signaling, [37]). It is interesting that the effect of *Nedd9* depletion on glucose uptake was extremely notable in the 344SQ and 531LN2 cell lines, both of which are highly metastatic and upregulate TGFβ signaling [38]; however, as these are murine lines, in contrast to the human lines used for much of the study, currently available data would also support the idea that this is a species-specific difference. An intriguing possibility is that endogenous levels of NEDD9 in progressing human tumors may be a determinant of response to therapeutic agents seeking to target the Warburg effect in human cancers—a point meriting further investigation.

## Materials and methods

### Cell culture and drug treatments

The A549 and H460 human NSCLC cell lines and HEK293 cells were obtained from the American Type Culture Collection (ATCC) and their identity verified by STR profiling. The murine cell lines (344SQ, 531LN2) were derived from *Kras*^*LA1/+*^ /*Trp53*^*R172HΔg/+*^ mice [38, 39]. For some experiments, cells were treated with vehicle (0.01% DMSO) or the SRC inhibitor dasatinib (MedChemExpress, Monmouth Junction, NJ, 50 nM for the A549 cell line, and 500 nM for the H460 cell line), or 2-Deoxy-D-glucose (2DG; MedChemExpress, Monmouth Junction, NJ, 10 mM for all cell lines).

### Animal samples used in the study

For Western blot and qRT-PCR analysis, we used tumor samples from B6.129 S/S4-*Kras*^*tm4Tyj/J*^ (*KP* mice) and B6.129 S/S4-*Kras*^*tm4Tyj/J*^ */Trp53*^*tm1Brn/J*^;*Nedd9*^*−/−*^ (*KPN* mice) described previously [17]. Briefly, tumor formation was induced with inhalation of adenovirus bearing Cre at 9 weeks of age and tumors developing within 10 weeks post inhalation. Surviving mice were euthanized at 39 weeks of age and tumors collected for subsequent analyses. All experiments involving mice were approved by the Institutional Animal Care and Use Committee (IACUC) of Fox Chase Cancer Center. Murine tumor samples were chosen randomly for subsequent analyses for each genotype.

### Gene depletion and overexpression

Transient transfection was performed using 30 nM NEDD9, HK1, HK2, and SCR negative control Smartpool siRNAs (with four pre-mixed siRNAs; Supplementary Table S1), from Dharmacon (Lafayette, CO) using Lipofectamine RNAiMAX (Thermo Fisher Scientific, Foster City, CA) transfection reagent, and harvested 48 h post-transfection for analysis.

For NEDD9 overexpression studies in NSCLC cells, cells were infected by lentivirus expressing the human NEDD9 cDNA [40]. Lentivirus was produced by transient co-transfection of HEK293T cells with a combination of packaging plasmid psPAX2 (cat #12260) and envelope plasmid pMD2.G (cat #12259) from Addgene, and a NEDD9 expression plasmid at a 1:1:2 concentration ratio using Mirus TransIT-LT1 transfection reagent (Mirus Bio LLC, Madison, WI). After 48 h, lentivirus-containing medium was collected, filtered, and added to NSCLC cell lines for 48 h prior to analysis. For transient overexpression in HEK293 cells, cDNAs encoding HK1 and HK2 with N-terminally fused GFP from Addgene (cat #21917 and #21920, respectively), and NEDD9 with an N-terminal hemagglutinin (HA) [41] were transfected using Mirus TransIT-LT1 transfection reagent (Mirus Bio LLC, Madison, WI), and cells collected after 24 h.

### Glycolytic function determination

A Seahorse flux analyzer (XF96 Extracellular Flux Analyzer; Seahorse Bioscience, North Billerica, MA, USA) was used in bioenergetic function assays [42] of cells with transiently depleted or overexpressed NEDD9 cells, using manufacturer’s protocols (https://www.agilent.com/en/products/cell-analysis/seahorse-analyzers). For some experiments, cells were treated with vehicle (0.01% DMSO) or dasatinib (50 nM for A549, and 500 nM for H460) 3 h prior to analysis. For treatment with EGF, cells were serum starved for 12 h and EGF was added 2 h prior to assay. Extracellular acidification rate (ECAR) was measured over 1 h after the cells had been conditioned in assay medium. A minimum of five replicates were used for each condition, and experiments were repeated at least three times. Data were processed using Wave Desktop software (Agilent Technologies, Santa Clara, CA); samples with negative values (suggesting a technical issue occurred during the measurement), were excluded from analysis.

### Measurement of glucose

Cells (2000 cells/well) were plated in 96-well cell culture plates in complete media. After 24 h, cells were transfected with siNEDD9 and SCR control siRNAs. After 48 h medium, aliquots were taken and cells were washed with PBS and lysed using 0.6 N HCL, followed by 1 M Tris base neutralization. Glucose detection reagent (Glucose-Glo kit, #J6021, Promega, Madison, WI) was added to measure glucose concentrations in cell lysates and medium samples according to the manufacturer’s instructions, and fluorescence was detected using a Perkin Elmer Envision Plate Reader. Assays were performed in 3 technical repeats and 3 biological repeats.

### Quantitative RT-PCR analysis

Total RNA was isolated from cell lines and tumor tissues using a Zymo Research Quick-RNA MicroPrep Kit (#R1050) and tested for quality on a Bioanalyzer (Agilent Technologies, Santa Clara, CA). RNA concentrations were determined with a NanoDrop spectrophotometer (Thermo Fisher Scientific, Waltham, MA), and analysis run using conditions and primers in Supplementary Table S2. For each sample, the values were averaged and standard deviation of data derived from two independent PCR experiments representing biological repeats.

### Western blot and IP analysis

For Western blot analysis, tumor tissues were lysed in T-PER buffer (ThermoScientific, Waltham, MA) and cultured cells were lysed in CelLytic MT Cell Lysis Reagent (Sigma-Aldrich, St. Louis, MO); concentrations were established using a Pierce BCA Protein Assay Kit (Thermo Scientific, Waltham, MA). Western blotting was performed using standard procedures and visualized using Luminata horseradish peroxidase (HRP) substrates (Classico, Crescendo and Forte, EMD Millipore, Burlington, MA) and Immun-Star AP Substrate (Bio-Rad Laboratories, Hercules, CA). Antibodies used are listed in Supp Table S3. Quantification was done using the NIH ImageJ software [43] with signaling intensity normalized to Ponceau S staining for the complete protein lane per sample.

For immunoprecipitation analysis cells were lysed in PTY buffer. Protein A/G plus agarose beads (sc-2003, Santa Cruz Biotechnology, Inc.) were incubated with primary antibody or IgG negative control (Supplementary Table S3) overnight, then 500 μg of protein lysates added and incubated overnight at 4 °C prior to bead collection and processing for western blotting.

### Statistical analysis

We used Mann-Whitney U two-tailed tests as appropriate for pairwise comparisons (all assumptions for the test were met). *P*-values <0.05 were considered as statistically significant and data presented as mean and S.E.M. No adjustment for multiple comparisons was applied. Analyses were performed using GraphPad Prism 8 (GraphPad Software, San Diego, CA). No statistical method was used to predetermine sample size. Sample sizes were estimated from previous experience and common knowledge of animal studies. All samples were deidentified when appropriate, and data was processed in a blinded manner.

## Supplementary information

Supplemental Figures and Tables

## Data Availability

The sources of cell lines, murine samples, and reagents used in this study are indicated in the respective “Material and Methods” subsections. Original IP/Western blot and qRT-PCR data from this study are available for download as a separate file. Researchers can obtain primary data for Seahorse and other functional assays reported in this study by contacting the corresponding author.
