# Supplementary material for: NEDD9 sustains hexokinase expression to promote glycolysis
Source: Oncogenesis. 2022 Apr 11;11(1):15. doi: 10.1038/s41389-022-00391-w (PMC9001639; doi:10.1038/s41389-022-00391-w)
Supplement: Supplementary file 1 — Supplemental Figures and Tables [file 41389_2022_391_MOESM1_ESM.pdf]

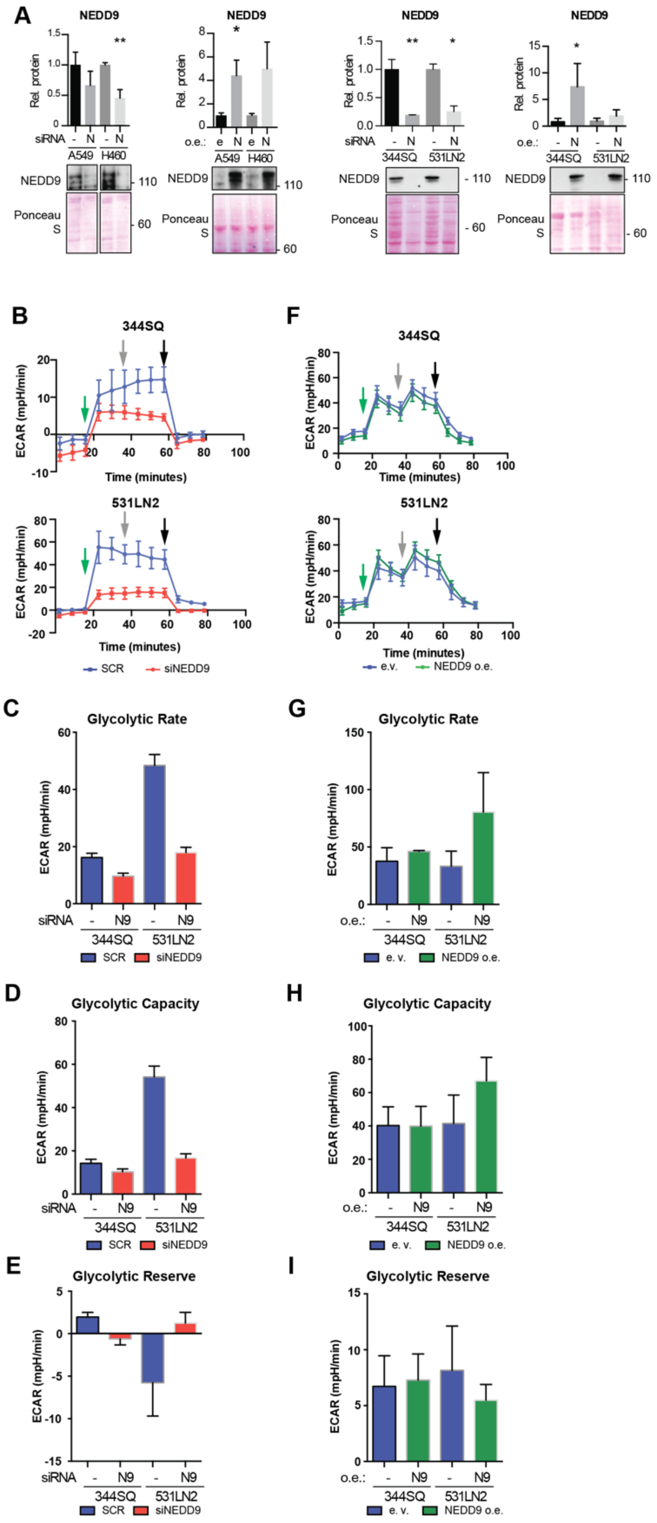

Deneka et al. Figure S1

Supp Figure S1. Depletion of NEDD9 reduces glycolysis. A. Western blot demonstrates effective depletion or overexpression of NEDD9, for experiments shown in Figure 1. For siRNA, control is indicated as (-), siRNA to reduce expression of NEDD9 as (N); for overexpression (o.e.), control is empty vector (e), versus NEDD9 overexpression (N) **B**. Seahorse results in 344SQ or 531LN2 cells treated with siRNA to *Nedd9* (red) or scrambled (blue). Arrows indicate addition of glucose (green), oligomycin (gray) and 2DG (black). **C-E**. Quantitation of results from **B** to demonstrate differences in glycolytic rate (**C**), glycolytic capacity (**D**), and glycolytic reserve (**E**). **F-I**. Experiments parallel those shown in **B-E**, but in conditions of 48 hours of NEDD9 overexpression (green).

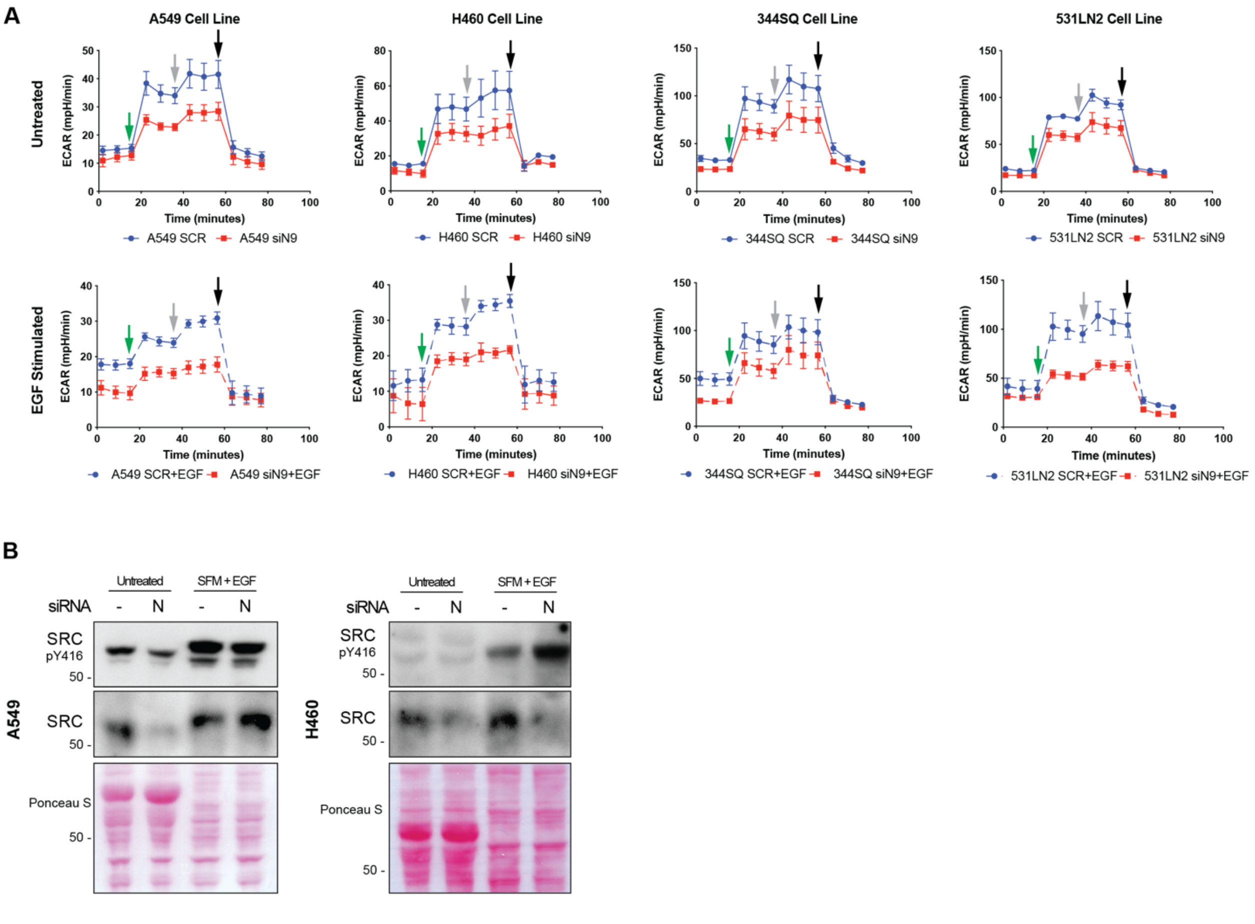

Deneka et al, Figure S2

Supp Figure S2. Stimulation of SRC with EGF does not rescue reduced glycolysis induced by NEDD9 depletion. A. Seahorse results in indicated cell lines transfected with siRNA to reduce NEDD9 (red) or scrambled (blue). Top row, cells are starved overnight (untreated); bottom row, analysis of cells after their overnight serum starvation followed by 2 hours of stimulation with EGF (125ng/ml). Arrows indicate addition of glucose (green), oligomycin (gray) and 2DG (black). **B**. Western blot analysis of phosphorylated (SRCpY416) and total SRC following transfection with siRNAs depleting NEDD9 (N) or Scrambled control (-) in control cells, or serum-starved cells stimulated with EGF.

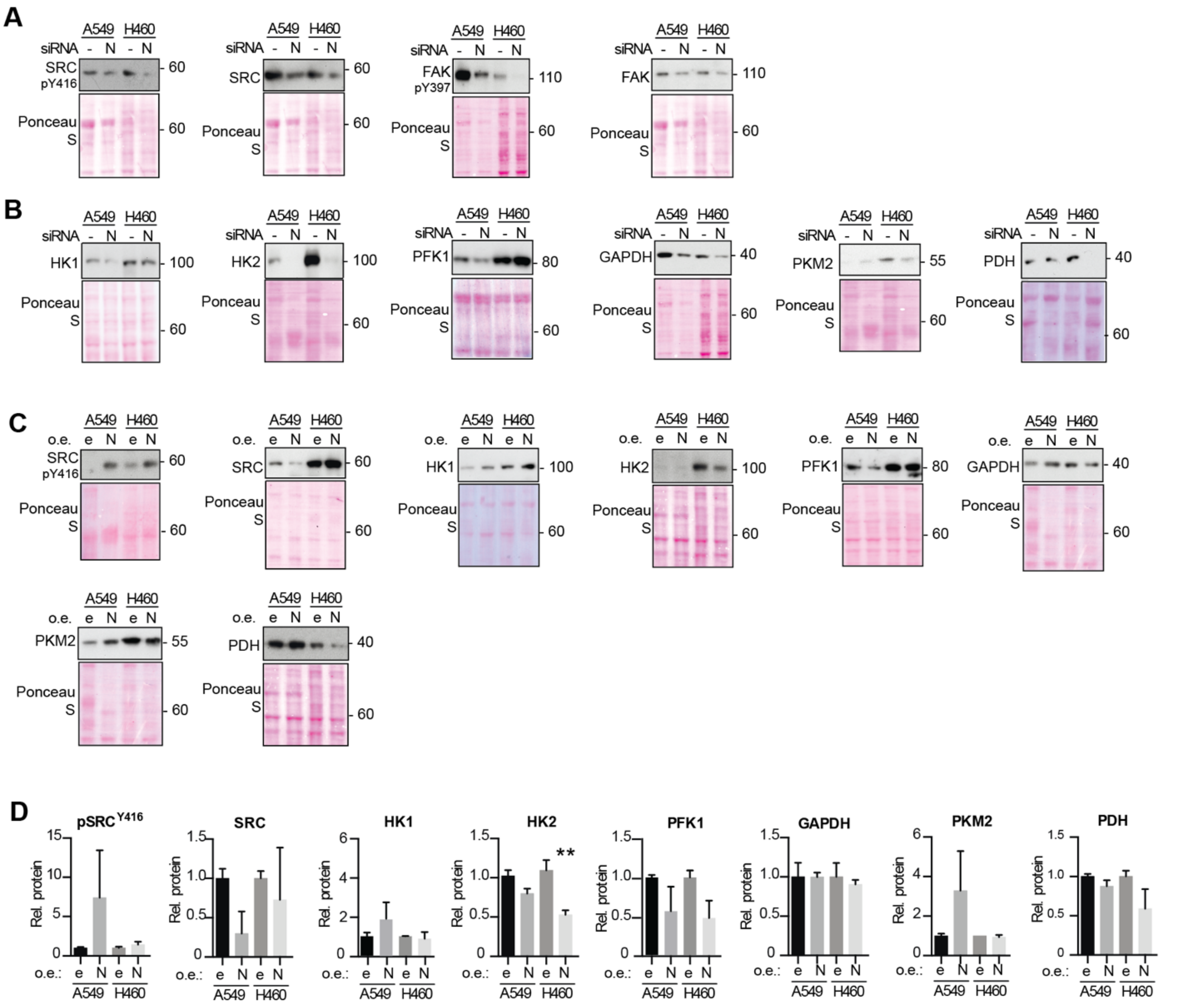

Deneka et al, Figure S3

Supp Figure S3. Depletion of NEDD9 in NSCLC cells reduces expression of hexokinases and other glycolytic enzymes; representative images. A-C. Representative Western blots from experiments paralleling those shown as averaged images in Figure 2. **A**. Western blot analysis of phosphorylated and total SRC and FAK following treatment with siRNAs depleting NEDD9 (N) or Scrambled control (-). **B**. Western blot analysis of glycolytic pathway proteins indicated, following NEDD9 depletion. **C**. Western blot analysis of proteins indicated, following NEDD9 overexpression (N), versus empty vector (e) control. Note, Western blots are performed on membranes that were cut in half to separately probe higher and lower molecular weight species, and/or were stripped and reprobed. Hence, Ponceau S images in some cases are shown with multiple Western blots, reflecting a common membrane of origin. **D**. Quantitation of Western blot analysis of proteins indicated in **C**, following NEDD9 overexpression (N), versus empty vector (e) control. All quantification is based on at least three independent experiments, with data normalized to Ponceau staining. *, $p<0.05$, **, $p<0.01$, ***, $p<0.001$, ****, $p<0.0001$ for all graphs.

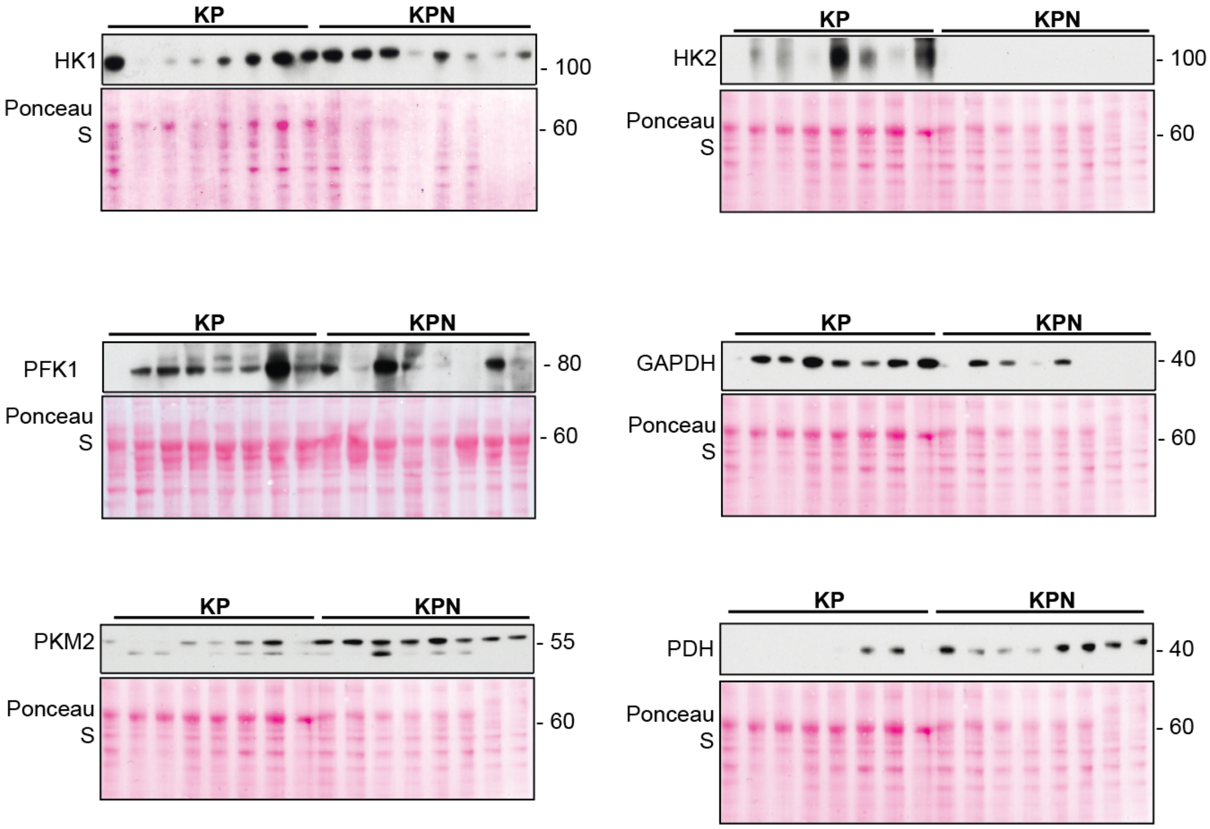

Deneka et al, Figure S4

Supp Figure S4. **Altered expression of glycolytic pathway enzymes in *KPN* versus *KP* murine NSCLC tumors: representative images**. Representative Western blots from experiments paralleling those shown as averaged images in Figure 3**.** Western blots shown indicated reduced (**A**) or increased (**B**) expression of expression of glycolytic pathway enzymes in *KPN* versus *KP* tumors. Note, Western blots are performed on membranes that were cut in half to separately probe higher and lower molecular weight species, and/or were stripped and reprobed. Hence, Ponceau S images in some cases are shown with multiple Western blots, reflecting a common membrane of origin.

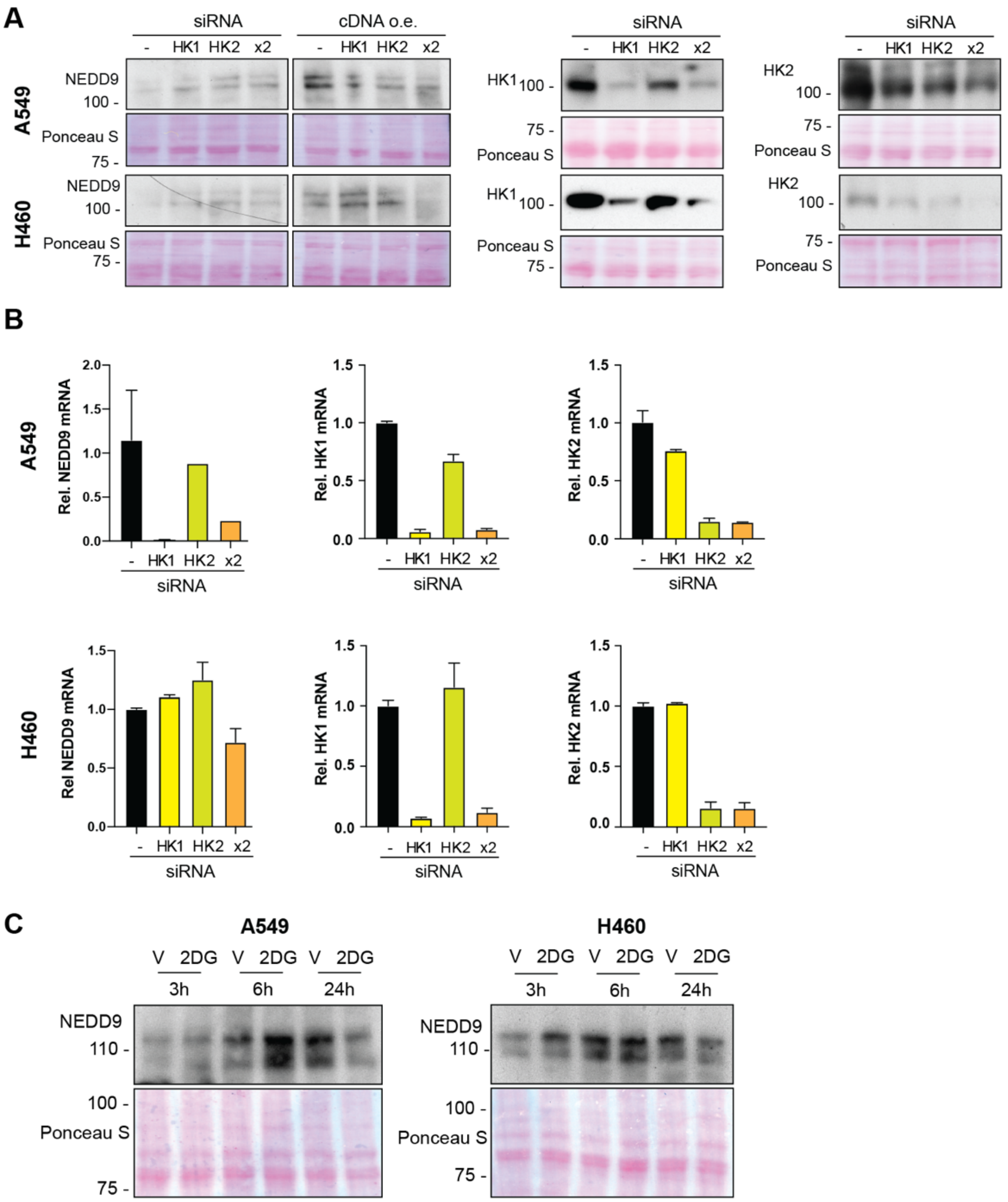

Deneka et al, Figure S5

Supp Figure S5. Hexokinase depletion and 2DG treatment induce NEDD9 expression. A-C. Representative Western blots from experiments paralleling those shown as averaged images in Figure 4C**. A.** Western blot analysis of NEDD9 expression in NSCLC cell lines with depletion (siRNA) or overexpression (o.e.) of control (-), HK1, HK2, or both (x2). **B**. RT-PCR results for NEDD9 or components of the glycolytic pathway, following siRNA depletion with scrambled siRNA (Scr) or indicated siRNAs. **C**. Western blot indicating NEDD9 expression in cells treated with vehicle (V) or 2DG for times indicated.

Supplemental Table 1. Smartpool siRNAs used in transient transfection experiments

Gene	Species	Cat #	Target sequence
NEDD9	H. sapiens	M-019466-02	5’-AGGAACUGGCCUUUCGCAA-3’<br>5’-CUACCAAAAUCAGGGAAUU-3’<br>5’-CCUCUGGACUGAUGCAGCA-3’<br>5’-CCAAGAACAAGAGGUAUAU-3’
NEDD9	M. musculus	L-059282-01-0005	5’-GCGAACAGCUCAAGACGAU-3’<br>5’-CAGGCAGCAUCUCGGGAUA-3’<br>5’-GGUUUGUGAUGGUCGCCAA-3’<br>5’-AGGAGGAGUUUGAGCGACA-3’
HK1	H. sapiens	L-006820-01-0005	5’-UCACCUGGACAAAGCGAUU-3’<br>5’-UACUAAGGGAUGCGAUAAA-3’<br>5’-GGUGAAAUCGUCCGCAACA-3’<br>5’-UGGGAGAGCUGGUUCGACU-3’
HK2	H. sapiens	L-006735-00-0005	5’-GAGUGGAGAUGCACAACAA-3’<br>5’-UGCAGAAGGUUGACCAGUA-3’<br>5’-GACGACAGCAUCAUUGUUA-3’<br>5’-CGGCUGCGCUCUACUAUUG-3’
SCR non-targeting		D-001810-10-20	5’-UGGUUUACAUGUCGACUAA-3’<br>5’-UGGUUUACAUGUUGUGUGA-3’<br>5’-UGGUUUACAUGUUUUCUGA-3’<br>5’-UGGUUUACAUGUUUCCUA-3’

Supplemental Table 2. Primers used for RT-PCR.

**Murine Primers**	
Hk1	TCACGTGGACAAAGGGATTC
	ACATCCAGGTCAAATTCCTCTC
Hk2	Seq in the middle of the amplicon:
	CAGTGGAACCCAGCTGTTTGACCAC
Pfk1	ACCAAACGTCGGGTGTTTAT
	ATGTTCAACATTCACCTGGAGA
Gapdh	TGATGGCAACAATCTCCACTTT
	AATGGTGAAGGTCGGTGTGAA
Pkm2	CAGGTGAAGGAGAAAGGCG
	TCAGGTCCTGGATGTCCTT
Pdh	ACACCAAGTTTATCCCTCCTAAC
	CCTAACCACATCCTGTCTATGC
**Human Primers**	
NEDD9	TGTGTTGCCTCTCAAACTCCTC
	GCCTGAGCAAGGAGCAGG
HK1	TTTGCACCATTGTCTCATTTCG
	AAACGCCGGGAATACTGTG
HK2	TTCTTGTCTCAGATTGAGAGTGAC
	CAGTGCACACCTCCTTAACA
PFK1	GGACCAGACAGATTTTGAGCA
	GTCAATCTCGTACTTGGCTAGG
PKM2	CTTTGCCATGAATGTTGGCAA
	ACGCATGGTGTTGGTGAA
PDH	CGATTAAGGCCACAGGATAAGT
	GGTACTCACCCACAATCCTAAC
GAPDH	Hs99999905_m1 assay from ThermoFisher

For analysis, RNA was reverse-transcribed using Moloney murine leukemia virus reverse transcriptase (Ambion-Thermo Fisher Scientific, Waltham, MA) and a mixture of anchored oligo-dT and random decamers (Integrated DNA Technologies, Coralville, IA). Aliquots of the cDNA were used to measure the expression levels of the genes using the primers or the commercial assay (Thermo Fisher) listed, with expression of genes of interest normalized to that of the housekeeping gene 36B4. Two reverse-transcription reactions were performed for each sample using either 100 or 25 ng of input RNA. Assays were used in combination with Power SYBR Green master mix (Thermo Fisher). All reactions were run on a 7900 HT sequence detection system (Applied Biosystems, Thermo Fisher Scientific Waltham, MA). Cycling conditions were 95°C, 15 min, followed by 40 (two-step) cycles (95°C, 15 s; 60°C, 60 s). Ct (cycle threshold) values were converted to quantities (in arbitrary units) using a standard curve (four points, four-fold dilutions) established with a calibrator sample. 36B4 was used as normalizer.

Supplemental Table 3. Antibodies used in the study

Antibodies used	Assay (IF, IHC, Western)	Source	Dilution
pY^{416}-SRC (#2101S)	Western	Cell Signaling (Beverly, MA)	1:1000
SRC (#2109S)	Western	Cell Signaling (Beverly, MA)	1:1000
pY397-FAK (#8556S)	Western	Cell Signaling (Beverly, MA)	1:1000
FAK (#3285S)	Western	Cell Signaling (Beverly, MA)	1:1000
HK1 (#2024S)	Western/IP	Cell Signaling (Beverly, MA)	1:1000/1:50
HK2 (#2867T)	Western/IP	Cell Signaling (Beverly, MA)	1:1000/1:50
PFK1 (#AF7687)	Western	R&D Systems (Minneapolis, MN)	1:1000
GAPDH (#5174S)	Western	Cell Signaling (Beverly, MA)	1:1000
PKM2 (#4053S)	Western	Cell Signaling (Beverly, MA)	1:1000
PDH (#3205S)	Western	Cell Signaling (Beverly, MA)	1:1000
NEDD9 (2G9)	Western	Abcam (Cambridge, United Kingdom)	1:1000
HA-Tag (#3724)	Western/IP	Cell Signaling (Beverly, MA)	1:1000/1:50
Anti-Rabbit IgG (GTX213110-01)	IP	GeneTex (Irvine, CA)	1:50
Secondary anti-rabbit HRP-conjugated antibody (#NA931)	Western	GE Healthcare (Little Chalfont, UK)	1:10000
Secondary anti-mouse HRP-conjugated antibody (#NA934)	Western	GE Healthcare (Little Chalfont, UK)	1:10000
Secondary anti-rabbit AP-conjugated antibody (#111-055-003)	Western	Jackson Immunoresearch Labs (West Grove, PA)	1:5000
Secondary anti-mouse AP-conjugated antibody (#115-035-003)	Western	Jackson Immunoresearch Labs (West Grove, PA)	1:5000
